# Faecal DNA to the rescue: Shotgun sequencing of non-invasive samples reveals two subspecies of Southeast Asian primates to be Critically Endangered species

**DOI:** 10.1038/s41598-020-66007-8

**Published:** 2020-06-10

**Authors:** Andie Ang, Dewi Imelda Roesma, Vincent Nijman, Rudolf Meier, Amrita Srivathsan, Rizaldi

**Affiliations:** 1 Raffles’ Banded Langur Working Group, Wildlife Reserves Singapore Conservation Fund, Singapore, Singapore 729826; 2 Department of Biology, Andalas University, Padang, West Sumatra, 25163 Indonesia; 3 Department of Social Sciences and Centre for Functional Genomics, Oxford Brookes University, Oxford, OX3 0BP United Kingdom; 4 Department of Biological Sciences, National University of Singapore, Singapore, Singapore 117543

**Keywords:** Biological techniques, Evolution, Genetics, Zoology

## Abstract

A significant number of Southeast Asian mammal species described in the 19^th^ and 20^th^ century were subsequently synonymized and are now considered subspecies. Many are affected by rapid habitat loss which creates an urgent need to re-assess the conservation status based on species boundaries established with molecular data. However, such data are lacking and difficult to obtain for many populations and subspecies. We document via a literature survey and empirical study how shotgun sequencing of faecal DNA is a still underutilized but powerful tool for accelerating such evaluations. We obtain 11 mitochondrial genomes for three subspecies in the langur genus *Presbytis* through shotgun sequencing of faecal DNA (*P*. *femoralis femoralis*, *P*. *f*. *percura*, *P*. *siamensis* cf. *cana*). The genomes support the resurrection of all three subspecies to species based on multiple species delimitation algorithms (PTP, ABGD, Objective Clustering) applied to a dataset covering 40 species and 43 subspecies of Asian colobines. For two of the newly recognized species (*P. femoralis*, *P. percura*), the results lead to an immediate change in IUCN status to Critically Endangered due to small population sizes and fragmented habitats. We conclude that faecal DNA should be more widely used for clarifying species boundaries in endangered mammals.

## Introduction

Human impacts on the environment have rapidly accelerated species extinction via habitat degradation and climate change and the recent report by the Intergovernmental Science-Policy Platform on Biodiversity and Ecosystem Services (IPBES) predicts that climate change has already affected the distribution of nearly half (47%) of land-mammals^1^. Protection is urgently needed but is hampered by the lack of data for a large number of mammal species, subspecies, and populations which face extinction^2–4^. A typical example is Asian primates for which 70% of the species are threatened^5^. Effective population management is needed but it requires a robust understanding of species numbers, boundaries, and distributions based on up-to-date information^6,7^. Unfortunately, this information is lacking for many rare, globally threatened, and elusive mammalian species. Many lack molecular data and collecting these data is difficult because invasive sampling yielding fresh tissues is usually not feasible.

This leaves only three alternative sources of DNA. The first is museum specimens, but the number of samples in museums tends to be small and many were collected in the 19^th^ or early 20^th^ century thus reflecting historic genetic diversity prior to extensive habitat loss. The second is tissue samples obtained from specimens that died of “natural causes” such as road accidents. The third source of genetic material is non-invasive samples such as hair and faeces. Faecal samples can be collected in good numbers during routine field surveys, which makes faecal samples particularly useful for data-deficient taxa that are in urgent need for re-assessment of species boundaries and distributions using molecular information^8^. Moreover, it is now straightforward to obtain complete mitochondrial genomes from such samples using shotgun sequencing^8–10^. Faecal samples contain a complex pool of DNA including that of the host. The host DNA is particularly informative because it reflects the current genetic diversity of the species. Faecal samples are nevertheless still an underappreciated source of information and many protocols for field research do not cover the collection of such samples^8^. Recent review by Srivathsan *et al*.^8^ estimates that it is possible to obtain data on host genetics and natural history for nearly 1,000 species of mammals in the next decade if faecal samples were to be collected routinely during field surveys and evaluated using metagenomics. In this study, we document the power of faecal metagenomics for testing the species boundaries in two species of Asian primates that are listed as Data Deficient on the IUCN Red List of Threatened Species.

Asian colobines (langurs and odd-nosed monkeys) are a diverse group of mammals, with 55 recognized species (87 spp.) belonging to seven genera (*Nasalis*, *Presbytis*, *Pygathrix*, *Rhinopithecus*, *Semnopithecus*, *Simias*, *Trachypithecus*)^11^; i.e., nearly half of all primate species in Asia are colobines. Unfortunately, many of these species are dependent on habitats that are quickly disappearing. Thus, nine species are already considered Critically Endangered, 23 are Endangered, and nine Vulnerable according to IUCN threat criteria^5^. This also applies to the genus *Presbytis* which is one of the most species-rich primate genera^12^. The 17 recognized species are found in the tropical rainforests of Sundaland, including the Malay Peninsula and the western Indo-Malay Archipelago^13,14^. Eleven species within *Presbytis* (*chrysomelas*, *comata*, *femoralis*, *frontata*, *hosei*, *melalophos*, *natunae*, *potenziani*, *rubicunda*, *siamensis*, and *thomasi*) were recognized in the last IUCN assessment^15^. The assessment predated the elevation of six subspecies to species (see Roos *et al*.^11^: *bicolor*, *canicrus*, *mitrata*, *sabana*, *siberu*, *sumatrana*) which suggests that many of the *Presbytis* taxa currently ranked as subspecies are in urgent need for re-assessment with molecular data. Unfortunately, these data are lacking for many subspecies and species which has serious consequences for the proper conservation assessment.

**Figure 1:**
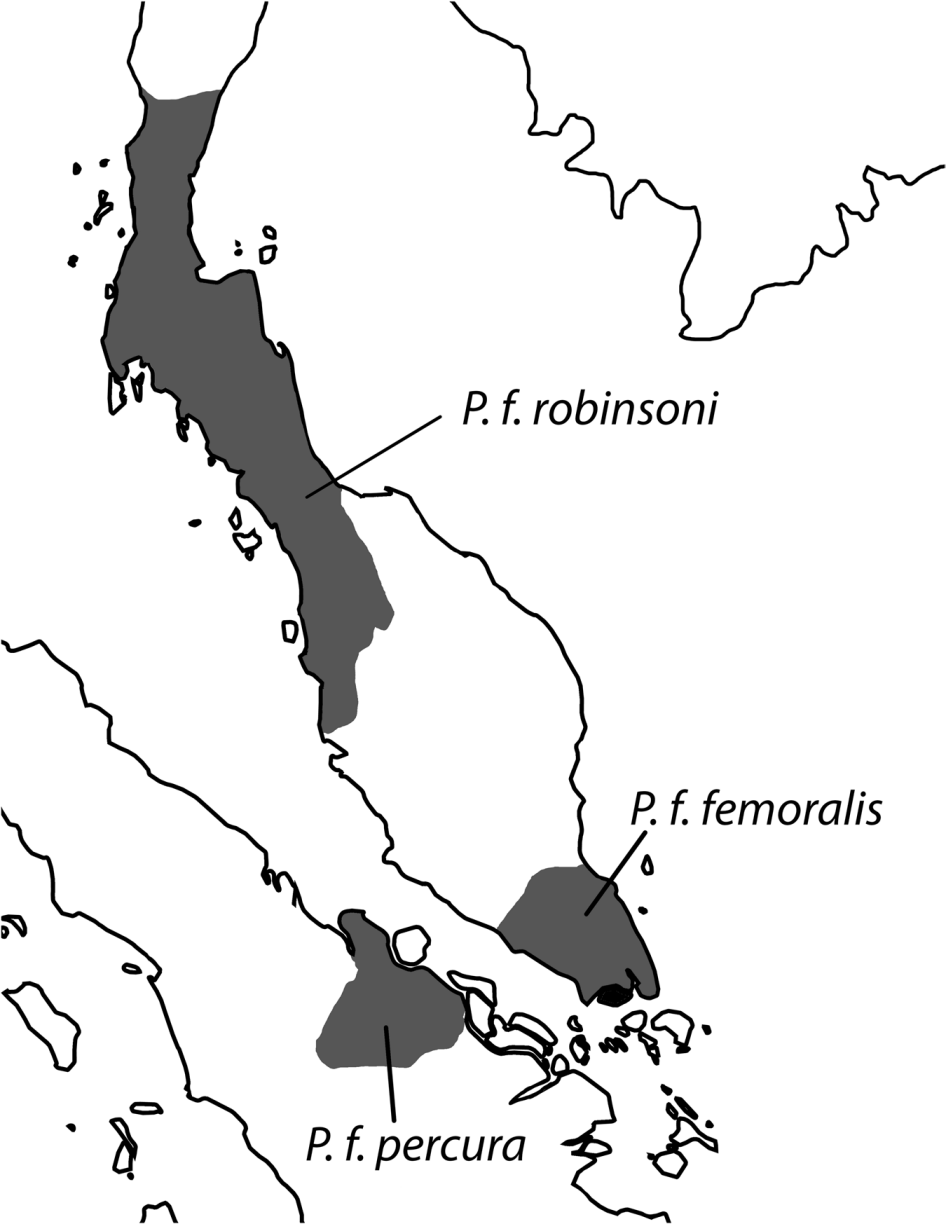
Distribution of three subspecies of *Presbytis femoralis*. Image: Ang Yuchen.

Meyer *et al*.^12^ presented the most comprehensive phylogenetic reconstruction of *Presbytis* which included 13 of the 17 recognized species, but the analysis was based on only two mitochondrial markers (cyt-b and d-*loop*). This study also covered the banded langur *P. femoralis* whose relationships had previously already been addressed by several studies*. Presbytis femoralis* is found on the Malay Peninsula and the island of Sumatra^16^ (Fig. 1). The species currently consists of three subspecies that were originally described as species because they are distinguishable based on a combination of morphological characters. Yet, many primatologists subsequently considered these characters insufficient for recognizing the three taxa as species (see Table 1).

**Table 1:** Morphological characters of three subspecies of *Presbytis femoralis*.

	*femoralis*	*percura*	*robinsoni*
Fur coat	Less dark; dusky greyish brown on the top of the head, the back, and the shoulders to the elbows	Less dark; upper parts of the head, the body, the feet, the hands, and the tail are black	Darkest; uniformly dark brown to black
Amount of white on body	The chin, a line down the chest and abdomen, the inside of the humeri from the axilla, and the inside of the thighs	The belly, a narrow line on the chest, the inner side of thighs extending to the heel, the inner side of arms from the axilla to the wrist, and the chin	The inner side of upper arms, lower abdomen following onto the inside of the thighs to the heel
Coloration of underparts	Pale of variable extent, but at least leaving the postumbilical area pale; the pale marking follows onto the inner side of the leg as a well-defined (femoral) stripe	Not uniform dark brown from umbilical region to chin; anterior margin of pale postumbilical region not sharply defined; outer side of thigh often with some trace of gray	Uniform dark brown from umbilical region to chin; anterior margin of pale postumbilical area sharply defined; outer side of thigh without trace of gray
Number of whorls on crown	A pair, with a long crest between, or one of the pair may be suppressed	One or (occasionally) a pair	One or a pair
Direction of hairs on chest	All directed backward	All directed backward	Directed more outward than backward on the sides of chest, and directed back in the midline of chest
Pale stripe on the underside of tail	Absent	Short and poorly developed	Very faint or absent
Condylo-basal length of skull	~65 mm	at least 70 mm	at least 70 mm
Mandible	~60 mm	~68 mm	~68 mm

References: Groves^16^; Martin^17^; Raffles^18^; Lyon^19^; Thomas^20^; Robinson and Kloss^21^; Weitzel *et al*.^22^; Miller^74^.

**Figure 2:**
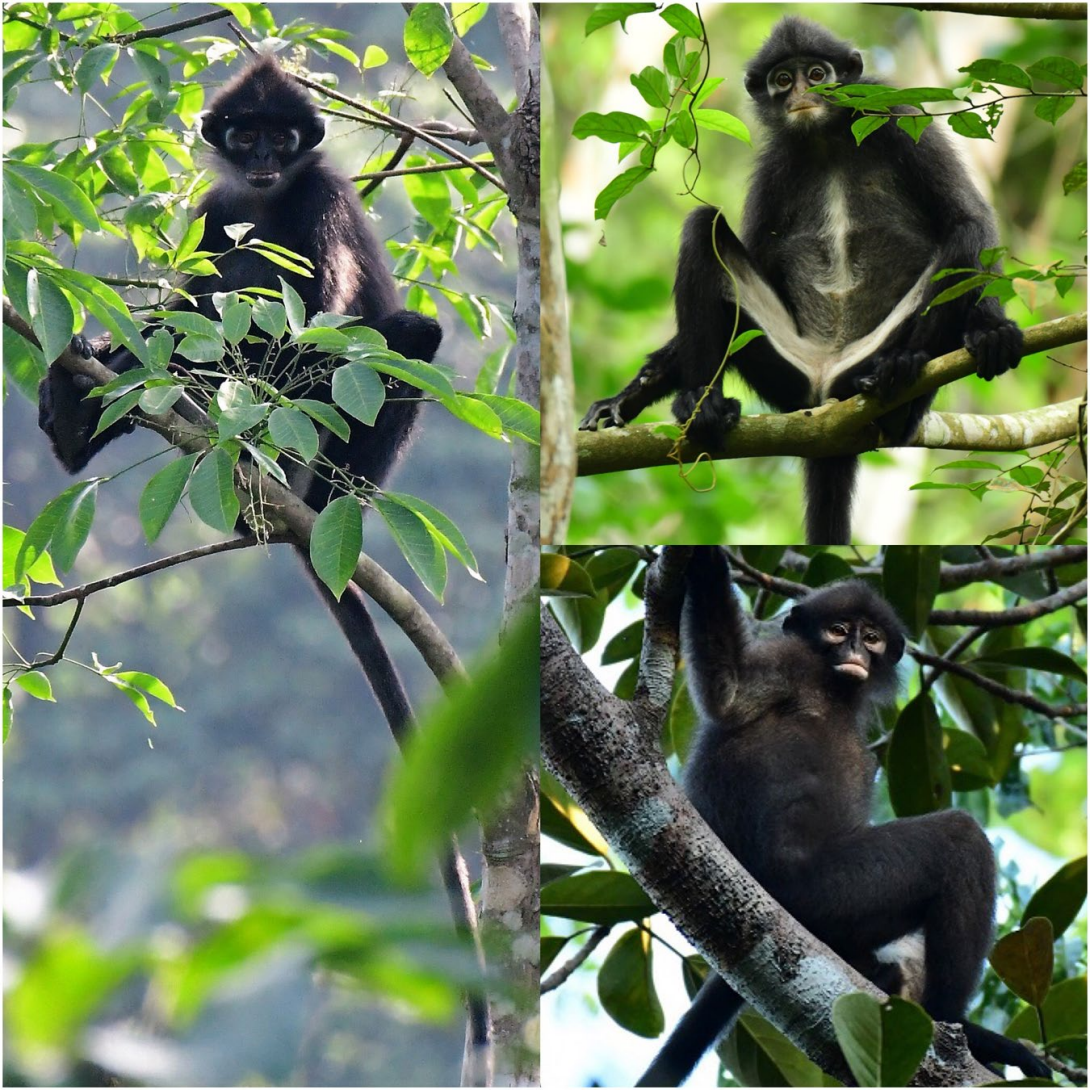
Three subspecies of *Presbytis femoralis*; clockwise from East Sumatran banded langur *P. f. percura* (1), Raffles’ banded langur *P. f. femoralis* (2), to Robinson’s banded langur *P. f. robinsoni* (3). Photos: Andie Ang.

The nominal species, *Presbytis femoralis*, was described by Martin (1838) based on specimens collected by Raffles (1821) from Singapore^17,18^ (Table 2). Raffles’ banded langur *P. f. femoralis* occurs in southern Peninsular Malaysia and Singapore. The second subspecies, the East Sumatran banded langur *P. f. percura* (Fig. 2), occurs only in eastern Sumatra and was described by Lyon (1908) based on specimens collected from near Siak Kecil River, Makapan, Kompei, Pulau Rupat, and Salat Rupat^19^. The third subspecies, Robinson’s banded langur *P. f. robinsoni*, was described by Thomas (1910) based on white phenotypic variants collected in Trang, southern Thailand^20–22^. However, typical *robinsoni* specimens are uniformly dark brown to black, with the inner side of the upper arms, lower abdomen following onto the inside of the thighs to the heel being white. *Presbytis f. robinsoni* is widespread and ranges from northern Peninsular Malaysia to southern Thailand and Myanmar.

**Table 2:** Taxonomic classifications of the type specimens of *femoralis*, *percura*, and *robinsoni* followed by later authors since their first descriptions (non-exhaustive).

Reference	*femoralis*	*percura*	*robinsoni*
Martin^17^	*Semnopithecus femoralis*	—	—
Lyon^19^	—	*Presbytis percura*	—
Thomas 1910^20^	—	—	*Presbytis robinsoni*
Elliot^75^	*Pygathrix femoralis*	*Pygathrix percura*	*Pygathrix robinsoni*
Miller^76^	*Presbytis femoralis*	—	*Presbytis keatii*
Miller^74^	*Presbytis femoralis*	*Presbytis percura*	*Presbytis keatii*
Pocock^77^	*Presbytis femoralis femoralis*	*Presbytis melalophos percura*	*Presbytis femoralis keatii*
Raven^78^	*Presbytis femoralis*	*Presbytis percura*	*Presbytis robinsoni*
Chasen^79^	*Pithecus femoralis femoralis*	*Pithecus femoralis percura*	*Pithecus femoralis robinsoni*
Hooijer^80^	*Presbytis melalophos femoralis*	—	—
Medway^81^	*Presbytis melalophos femoralis*	—	*Presbytis melalophos robinsoni*
Thorington and Groves^82^	*Presbytis melalophos femoralis*	*Presbytis melalophos percura*	*Presbytis melalophos robinsoni*
Wilson and Wilson^83^	—	*Presbytis femoralis percura*	—
Medway^84^	*Presbytis melalophos femoralis*	—	*Presbytis melalophos robinsoni*
Brandon-Jones^85^	*Presbytis femoralis femoralis*	*Presbytis femoralis percura*	*Presbytis femoralis robinsoni*
Napier^86^	*Presbytis melalophos femoralis*	*Presbytis melalophos percura*	*Presbytis melalophos robinsoni*
Weitzel *et al*.^22^	*Presbytis femoralis femoralis*	—	*Presbytis femoralis robinsoni*
Aimi and Bakar^87^	—	*Presbytis femoralis percura*	—
Oates *et al*.^13^	*Presbytis melalophos femoralis*	*Presbytis melalophos percura*	*Presbytis melalophos robinsoni*
Groves^16^	*Presbytis femoralis femoralis*	*Presbytis femoralis percura*	*Presbytis femoralis robinsoni*
Brandon-Jones *et al*.^88^	*Presbytis femoralis femoralis*	*Presbytis femoralis percura*	*Presbytis femoralis robinsoni*
Md.-Zain^89^	*Presbytis melalophos femoralis*	—	*Presbytis melalophos robinsoni*
Meyer *et al*.^12^	*Presbytis femoralis femoralis*	—	*Presbytis femoralis robinsoni*
Vun *et al*.^27^	*Presbytis melalophos femoralis*	—	*Presbytis melalophos robinsoni*
Roos *et al*.^11^	*Presbytis femoralis femoralis*	*Presbytis femoralis percura*	*Presbytis femoralis robinsoni*
Abdul Latiff *et al*.^28^	*Presbytis neglectus neglectus*	*Presbytis femoralis percura*	*Presbytis femoralis robinsoni*
This study	*Presbytis femoralis*	*Presbytis percura*	*Presbytis robinsoni*

*Presbytis femoralis*, consisting of all three subspecies, is listed as Vulnerable in the most recent IUCN Red List assessment, with the nominate subspecies considered Endangered because the known populations are restricted to small and isolated patches of forest. In addition, one population from Singapore showed low genetic variability^10,23^. *Presbytis f. robinsoni* is considered Near Threatened, while the least known and least studied subspecies is *P. f. percura* was considered Data Deficient^24^. Genetic data suggest that at least *P. f. femoralis* and *P. f. robinsoni* are different species^25,26^ which is also in agreement with the aforementioned morphological characters. However, resolving all subspecies-level boundaries within banded langurs required data for *P. f. percura*.

For several reasons the subspecies boundaries within *P. femoralis* remain poorly understood. The main problem is the lack of molecular data for *P. f. percura*. However, even if the data were available, a comprehensive analysis would still be difficult because the sequence data from three published analyses were not submitted to public sequence repositories (cyt-b, 12 S rDNA, and d-*loop*)^27–29^. This means that currently the only publicly available molecular data are for *P. f. femoralis* from the type locality in Singapore (KU899140)^10^ and *P. f. robinsoni* from Redang Panjang, Malaysia (DQ355299)^30^. Note that the genome DQ355299 was initially submitted to Genbank as *P. melalophos* but it was later clarified by Meyers *et al*.^12^ that it is from *P. f. robinsoni*. Fortunately additional molecular data can be reconstructed based on a table published in Abdul-Latiff *et al*.^28^ that lists the variable d-*loop* sites for several species and subspecies (see Nijman^26^). Another complication related to resolving species limits within *P. femoralis* is confusing nomenclatural changes. Abdul-Latiff *et al*.^28^ proposed to replace the type species *P. femoralis* (Martin 1838) with a junior synonym (*P. neglectus neglectus* Schlegel 1876)^31^ without considering the detailed information in Low and Lim^32^ that explains why Martin is the author of the name *femoralis* and Singapore the type locality of the species. Abdul-Latiff *et al*.^28^’s study furthermore violated its own proposed nomenclatural changes by retaining *P. f. percura* and *P. f. robinsoni* (see Nijman^26^).

Here, we solve these problems by providing the first mitogenomes of *P. f. percura* and thus addressing the taxonomic status of all three subspecies of banded langurs. We also obtain the first mitogenome for the Riau pale-thighed langur *P. siamensis* cf. *cana* from Sumatra which helps with resolving subspecies limits within this species. Lastly, we provide an updated dated phylogenetic tree for Asian colobines based on mitochondrial genomes and survey the mammal literature to illustrate that faecal DNA is currently still an underutilized source of genetic information.

## Results

### Survey of zoological record

In order to investigate to what extent faecal samples have been used in addressing taxonomic problems, we surveyed the literature as captured in Zoological Record. We retrieved 1,852 articles that mentioned faecal samples, but apparently only a subset of 43 articles addressed systematic/taxonomic issues because they were also classified under Systematics/Taxonomy. Inspection of these records revealed only two studies that used faecal DNA for resolving species limits^33,34^.

### Sequence data

Illumina sequencing of faecal metagenomes yielded 60.3–69.7 million sequences for each sample from Sumatra (*Presbytis femoralis percura:* ESBL1–8, *P. siamensis* cf*. cana*: Pres2; Table 3). The data were combined with the Hi-Seq data for six samples from Singapore for *P. f. femoralis* (BLM1–6) sequenced as part of study by Srivathsan *et al*.^10^. All data were quality trimmed using Trimmomatic^35^ and complete mitochondrial genomes were obtained (*P. f. femoralis:* 16,548 bp, *P. f. percura*: 16,548 bp, *P. s*. cf. *cana:* 16,558 bp). Human contamination was found to be negligible (0.06% of mitochondrial reads) and did not impact the reconstruction of the mitochondrial genomes (see methods). One sample of *P. f. percura* ESBL_7 had low average coverage of <5X for the mitochondrial genome and was not analysed further. Data corresponding to the mitochondrial genomes are available as Supplementary Materials 2 and 3.

**Table 3:** Summary of sequencing data and mitochondrial genomes obtained from eleven *Presbytis* langurs from Singapore and eastern Sumatra.

Sample ID	Location	Organism	# Raw/trimmed reads (millions)/Gbp	Average mitochondrial coverage (X)/# mapped reads/mean length	Accession #
ESBL_1b	Kampar, Riau	*Presbytis femoralis percura*	60.33/58.81/18	25.205/2874/147.9	MN496093
ESBL_5	Bengkalis, Riau	*Presbytis femoralis percura*	63.38/61.74/19	5.155/587/148.1	MN496095
ESBL_6a	Bengkalis, Riau	*Presbytis femoralis percura*	67.04/66.61/20	8.721/990/148.6	MN496096
ESBL_8b	Bengkalis, Riau	*Presbytis femoralis percura*	69.69/67.98/21	13.275/1513/148	MN496094
Pres2	Kampar, Riau	*Presbytis siamensis* cf. *cana*	67.37/65.69/20	9.358/1054/147.8	MN496097
BLM1	Central Catchment Nature Reserve, Singapore	*Presbytis femoralis femoralis*	107.68/92.35/16	21.794/4764/75.7	MN496088
BLM2	Central Catchment Nature Reserve, Singapore	*Presbytis femoralis femoralis*	72.66/60.18/11	19.716/4307/75.8	MN496091
BLM3	Central Catchment Nature Reserve, Singapore	*Presbytis femoralis femoralis*	85.96/74.05/13	15.029/3282/75.8	MN496089
BLM4	Central Catchment Nature Reserve, Singapore	*Presbytis femoralis femoralis*	66.99/56.88/10	37.606/8220/75.7	MN496090
BLM5	Central Catchment Nature Reserve, Singapore	*Presbytis femoralis femoralis*	68.19/56.15/10	104.315/22796/75.7	KU899140
BLM6	Central Catchment Nature Reserve, Singapore	*Presbytis femoralis femoralis*	76.44/65.11/12	12.708/2781/75.6	MN496092

### Species delimitation

Pairwise comparison of cyt-b, hypervariable region HV1 of the d-*loop* and mitochondrial genomes (CDS + rDNA+d-*loop*) revealed minimum genetic divergence of 7.1%, 6.1% and 5.3% between *P. f. femoralis* and *P. f. percura*. On the other hand, the minimum pairwise distance between either of these taxa with *P. f. robinsoni* is 6.0% for HV1, 10.3% for cyt-b and 7.6% across the mitochondrial genome. For the two subspecies of *P. siamensis*, we were only able to compare HV1 sequences. The HV1 sequence of *P. s*. cf. *cana* has a 11.1% divergence from *P. s. siamensis* and 5.1% from *P. melalophos* (KY117602), while cyt-b and complete mitochondrial genomes show divergence of 2.8% and 2.5% between sequences from *P. s*. cf. *cana* and *P. mitrata/P. melalophos*. Overall, these results suggest that *P. s*. cf. *cana* represents a genetically distinct *Presbytis* lineage.

The high genetic differentiation leads to the recognition of several species based on multiple species delimitation algorithms such as Poisson Tree Processes (PTP)^36^, Automated Barcode Gap Discovery (ABGD)^37^ and Objective Clustering^38^. PTP consistently split *P. f. femoralis* and *P. f. percura* into different molecular Operational Taxonomic Units (mOTUs) across the three datasets examined (1. Asian colobine mitogenome, 2. *Presbytis* mitogenome+cyt-b + HV1, 3. *Presbytis* HV1-only) (Supplementary Figs. 1–3). ABGD and Objective Clustering (thresholds 2–4%) similarly assigns these two subspecies to different species across the three different datasets and a range of parameters (Table 4). For ABGD, these subspecies would only lump if unusually high priors for intraspecific divergences were used (priors > =0.0215). These parameters are not likely to be appropriate because they also led to the collapse of many recognized *Presbytis* species into a single mOTU. All three species delimitation methods placed *P. f. robinsoni* as a distinct species from *P. f. femoralis* and *P. f. percura*. Lastly, *P. s*. cf. *cana* was placed as a distinct species using PTP and ABGD unless inappropriately high priors for intraspecific divergence are used in ABGD. Objective Clustering based on HV1 also identified *P. s*. cf. *cana* as a distinct species. However, the mitogenome and cyt-b datasets lumped *P. s*. cf. *cana* with *P. melalophos* and *P. mitrata* at 3% and 4% thresholds.

**Table 4:**
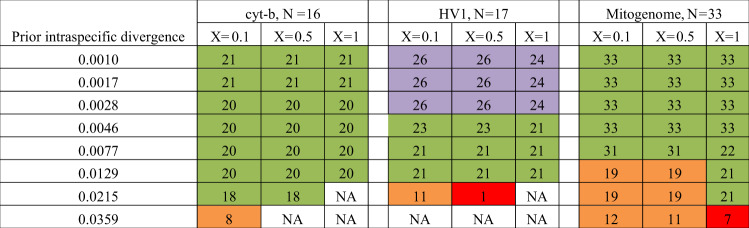
Summary of results of ABGD-based species delimitation.

Values represent number of molecular Operational Taxonomic Units (mOTUs). N represents number of species (include those resurrected in this study), X the slope parameter. Colours: green: all resurrected species are valid. Purple: Supports splitting of the three subspecies of *Presbytis femoralis* and splitting of *P. siamensis* cf. *cana*. However, some sequences of *P. f. femoralis* from Peninsular Malaysia are split into multiple mOTUs. Orange: Resurrection of all three subspecies of *P. femoralis* is valid, but *P. s*. cf. *cana* is lumped with other *Presbytis* species. Red: Not valid.

Note that we observed further species-level splitting when species delimitation was based on only HV1 data for the subspecies of *P. femoralis*. This dataset included sequences for multiple individuals of *P. f. femoralis, P. f. robinsoni* and *P. s. siamensis* from the Malay Peninsula (reconstructed in Nijman^26^, based on Abdul Latiff *et al*.^28^). At low prior intraspecific divergences, ABGD split some haplotypes of *P. f. femoralis* from the Malay Peninsula into separate mOTUs from other haplotypes of the same subspecies from the same region. These, however, consistently grouped together as single mOTU at higher thresholds. Similarly, PTP based analyses of only HV1 data split haplotypes of *P. f. robinsoni* into multiple mOTUs (Fig. S2) while ABGD consistently placed them as a single species.

### Mitochondrial phylogeny of Asian colobines and genus *Presbytis*

**Figure 3:**
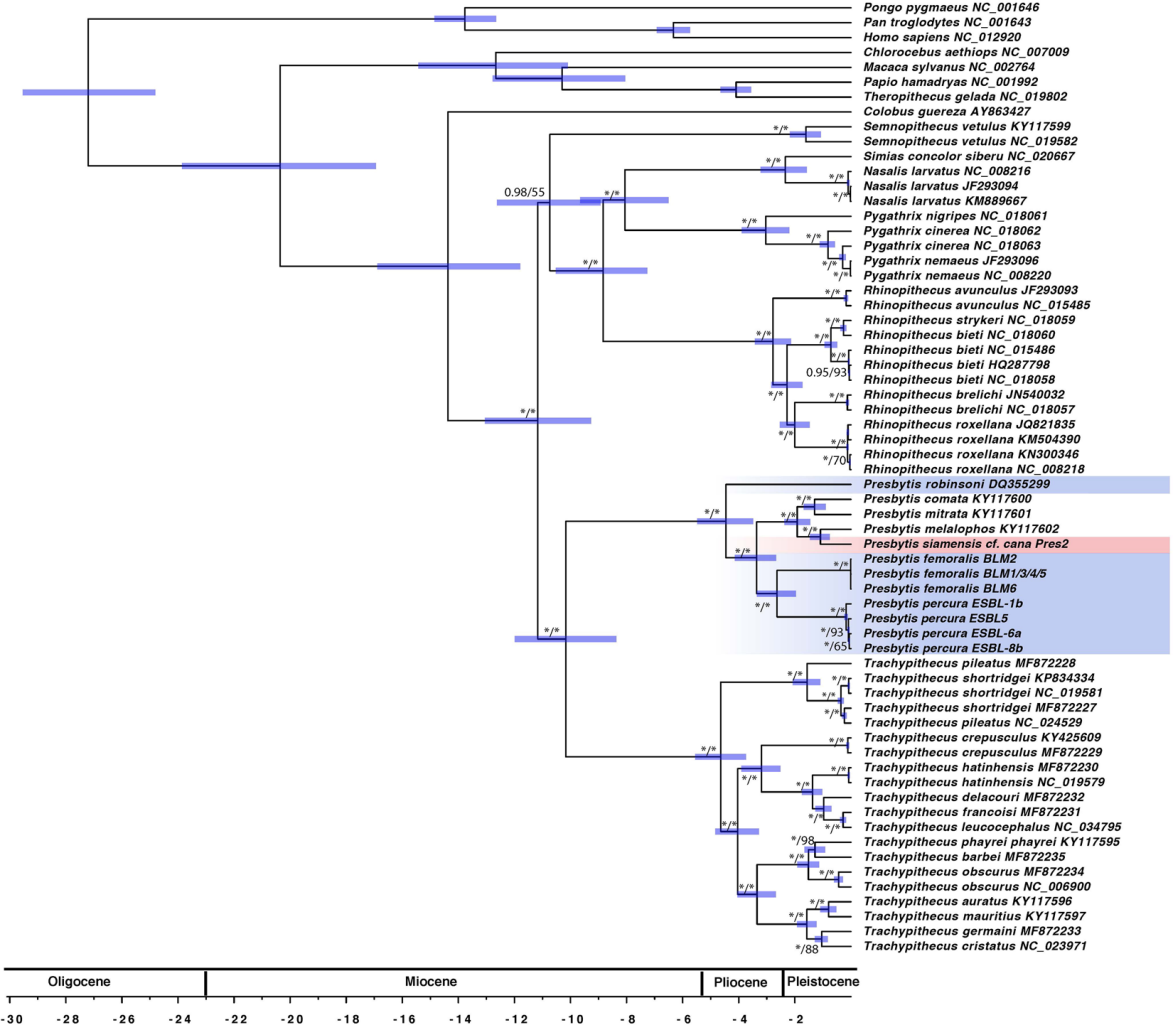
Dated phylogeny of Asian colobine primates based on mitochondrial genomes. The values at nodes represent posterior probability (codon partitioning)/ML bootstrap support for relationships between Asian colobines. Values are omitted if both BI and ML support values are <0.7/70, while * represents support of 1/100. The bars represent the 95% confidence intervals for divergence times estimates. KU899140 is here referred to as BLM5.

The phylogenetic reconstruction based on mitochondrial genomes of the Asian colobine dataset revealed that *P. femoralis* is polyphyletic (Fig. 3). The reconstructions based on Maximum Likelihood (ML) and Bayesian Inference (BI) are congruent and reveal that *P. f. femoralis* and *P. f. percura* are sister taxa. Divergence time estimates dated the split of *P. f. femoralis* and *P. f. percura* at 2.6 Mya (CI: partitioning by codon: 1.96–3.35 Mya, partitioning by gene 1.90–3.37 Mya (Supplementary Fig. S4)). This clade is sister to a clade comprising of *P. mitrata, P. comata, P. siamensis* cf. *cana*, and *P. melalophos*. *Presbytis f. robinsoni* diverged from these species at 4.5 Mya (CI: partitioning by codon: 3.49–5.48 Mya, partitioning by gene 3.46–5.62). Overall, the mitochondrial phylogeny reveals high support for a clade comprising of *Presbytis* and *Trachypithecus* as well as the relationships within a clade consisting of four genera (((*Simias* + *Nasalis*)+*Pygathrix*)+*Rhinopithecus*). Only the placement of *Semnopithecus* remains uncertain, as revealed by low support for its relationship to the clade comprising of *Nasalis*, *Simias*, *Pygathrix* and *Rhinopithecus* on the ML tree. This result is different from Wang *et al*.^39^, who found high support for a sister group relationship of *Semnopithecus* to all remaining genera of Asian colobines. However, a combined analysis of nuclear and mitochondrial data placed *Semnopithecus* differently thus suggesting our mt-genome phylogeny correctly reflects that the placement of *Semnopithecus* remains uncertain.

**Figure 4:**
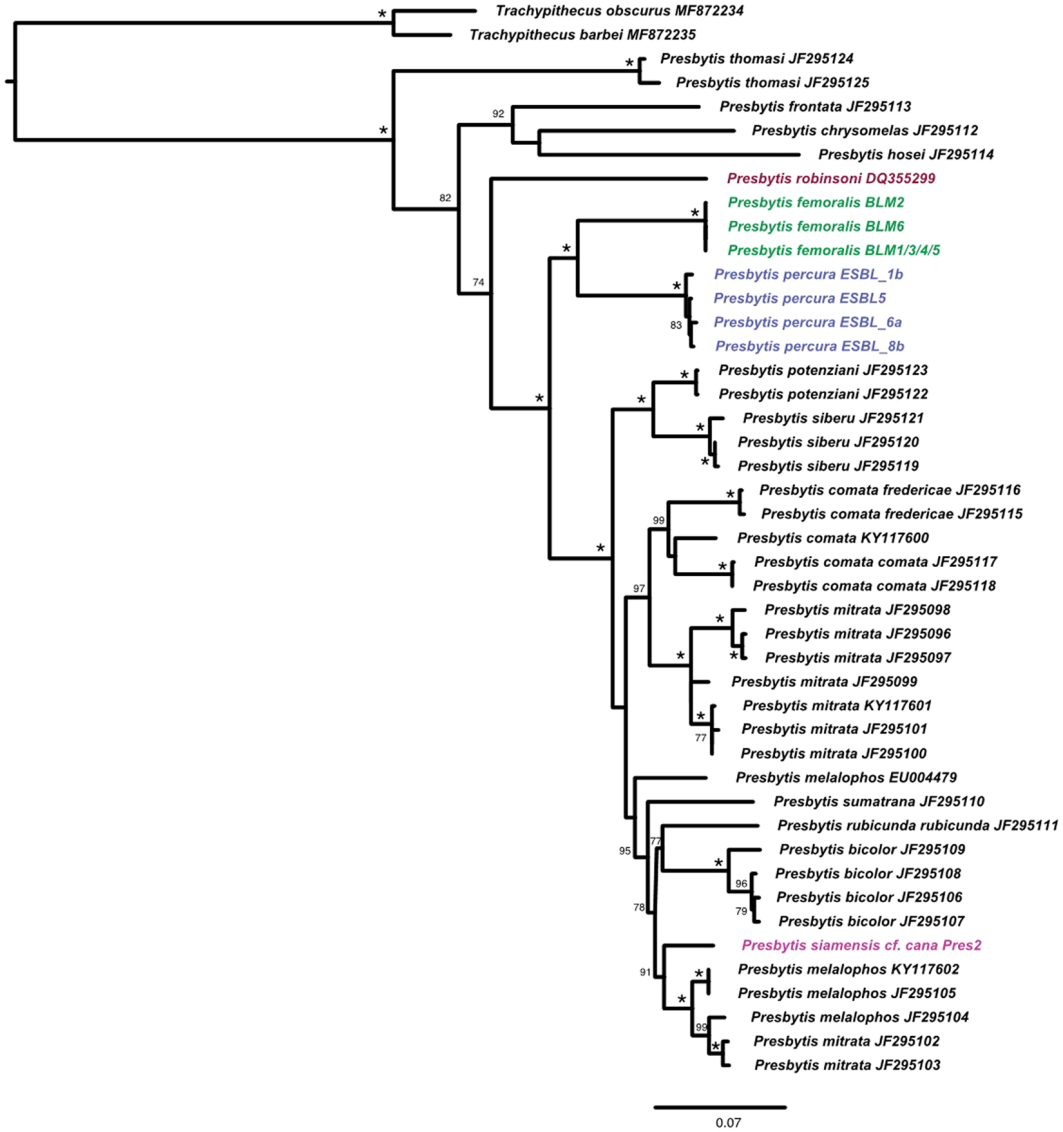
ML reconstruction of relationships between *Presbytis* species based on mitogenome+cyt-b + HV1 dataset. Node values represent bootstrap support, values <70 are excluded, while node support of 100 is represented by *. KU899140 is here referred to as BLM5.

With regard to the phylogenetic relationships within *Presbytis*, our results on *Presbytis* mitogenome+cyt-b + HV1 dataset (Fig. 4) are largely consistent with the reconstruction by Meyer *et al*.^12^. The only differences are as follows: low support for a clade comprising of *P. comata, mitrata, melalophos, bicolor, sumatrana, rubicunda* and *P. siamensis* cf. *cana* but resolution for *P. rubicunda, melalophos, mitrata* and *bicolor* which formed a trichotomy in Meyer *et al*.^12^. Here, we found *P. rubicunda* to be sister to *P. bicolor. Presbytis f. femoralis* and *P. f. percura* remain sister taxa. Both taxa combined are more closely related to *P. mitrata, P. comata* and several other taxa of *Presbytis* than *P. f. robinsoni*. The split between *P. f. femoralis* and *P. f. percura* is again deeper than for most recognized taxa of *Presbytis*. Divergence estimates based on cyt-b for this taxon set revealed deeper divergence times as compared to mitochondrial genomes, but with overlapping confidence intervals. *Presbytis f. femoralis* and *P. f. percura* split 2.93 Mya (2.09–3.78 Mya) (Supplementary Fig. S5), while *P. f. robinsoni* diverged from the clade comprising of *P. femoralis, potenziani, mitrata, melalophos, bicolor, sumatrana* and *P. siamensis* cf. *cana* at 5.47 Mya (CI: 4.28–6.66 Mya).

## Discussion

The species limits of many Southeast Asian mammal taxa remain unclear which interferes with a conservation assessment at a time when many populations, subspecies, and species face extinction. We here demonstrate how such taxonomic uncertainty can be addressed rapidly through shotgun sequencing of faecal DNA. We document the power of the approach by studying langur species in the genus *Presbytis* Eschscholtz, 1821 which continue to undergo many taxonomic changes that significantly affect the conservation status of these taxa. At one point all Asian langurs and leaf monkeys in *Presbytis*, *Semnopithecus*, and *Trachypithecus* were included in *Presbytis* and only five widespread species were recognized (*P. aygula*, *P. melalophos*, *P. frontata*, *P. potenziani*, *P. rubicunda*)^40–42^. This has dramatically changed over the last 20 years and currently three genera and 45 species are recognized (17 spp. in *Presbytis*; eight spp. in *Semnopithecus*; 20 spp. in *Trachypithecus*)^5,11^. Many of these changes in species boundaries were based on genetic data which allowed for the application of explicit species delimitation methods^27,39,43^. These new data and analyses revealed that many taxa that were initially described as species and later downgraded to subspecies diverged well before the Pleistocene and should be recognized as species; i.e., the morphological characters that were used for the initial species descriptions were appropriate for the delimitation of species and the subsequent lumping was not justified.

### Resurrection of *Presbytisfemoralis*, *P. percura* and *P. robinsoni*

Based on multiple species delimitation methods, high genetic divergence, placement in the mitochondrial phylogenies, as well as distinct morphological differences, we here resurrect the three species of *P. femoralis* from their current subspecific status (Table 2). The newly circumscribed Raffles’ banded langur *P. femoralis* is now only known from southern Peninsular Malaysia (states of Johor and Pahang) and Singapore. The East Sumatran banded langur *P. percura* only occurs in Riau Province of east-central Sumatra. Lastly, Robinson’s banded langur *P. robinsoni* has the widest distribution and ranges from northern Peninsular Malaysia (states of Kedah and Perak) through southern Thailand (provinces of Surat Thani, Phetchaburi, and Prachuap Khiri Khan) to southern Myanmar (Tanintharyi Region). These changes to species status mean that *Presbytis* now comprises 19 species.

A >5% genetic difference and divergence estimates of 2.6–2.9 Mya between *P. femoralis* and *P. percura* suggest that these two species diverged prior to the Pleistocene, while several other species of *Presbytis* originated more recently. These results are particularly intriguing because the changing sea levels during the Pleistocene would have increased connectivity between the land masses of Sumatra and Malay Peninsula. However, the Malacca Straits River flowing northwards with tributaries in what is now Sumatra and the Malay Peninsula^44^ may have been a substantial barrier between *P. femoralis* and *P. percura*, as it would have been significantly wider than the rivers that currently form geographic barriers between some of the *Presbytis* species in Sumatra. Furthermore, it has been argued that the land bridge between Sumatra and the Malay Peninsula had coarse sandy and/or poorly drained soils which may have limited plant growth in central Sundaland. Unsuitable vegetation may have acted as a dispersal barrier for rainforest plants and animals^45^. These barriers would have kept the langur populations separate; i.e., it remains unclear whether *P. femoralis* and *P. percura* would have formed a hybrid zone if they had encountered each other. Note that it is known that currently recognized primate species in *Trachypithecus* that radiated ~0.95–1.25 Mya can interbreed^43,46^, but the genetic divergence between *P. femoralis* and *P. percura* is considerably higher.

### Conservation status of *P. femoralis*, *P. percura*, and *P. robinsoni*

In the most recent IUCN Red List assessment (unpublished data from a Red List re-assessment in 2015), *Presbytis femoralis* (comprising *femoralis*, *percura* and *robinsoni*) was listed as Vulnerable (A2cd A3cd A4cd: population size reduction of at least 30% over three generations based on a decline in area of occupancy, extent of occurrence and habitat quality, and actual or potential levels of exploitation). As part of this assessment the status of the three subspecies were also evaluated. *Presbytis f. femoralis* was considered Endangered (A2cd A3cd A4cd), *P. f. percura* Data Deficient, and *P. f. robinsoni* Near Threatened. With their resurrection to species rank, the conservation status of each of the taxa requires re-assessment. *Presbytis femoralis* has a small global population size which continues to decline mainly due to habitat loss. There are 60 individuals (48 mature individuals) in the Singapore population of *P. femoralis*^47^. There are no precise population estimates available for the conspecifics in the Malaysian states of Johor and Pahang, but it is believed that only a few hundred individuals remain (see Abdul-Latiff *et al*.^28^); i.e., the overall population of *P. femoralis* could well be <250 mature individuals. Furthermore, the extensive habitat loss especially to industrial-scale oil palm plantations in southern Peninsular Malaysia is unlikely to cease in the near future (see Shevade *et al*.^48^; Shevade and Loboda^49^). Hence, based on a small population size and decline, we propose to list *P. femoralis* as Critically Endangered (C2a(i): <250 mature individuals, continuing population decline, and ≤50 mature individuals in each subpopulation).

*Presbytis percura* is only found in a number of isolated forests and faces extinction in the wild based on large-scale forest loss in Riau Province^50^. Riau experienced the highest rate of deforestation in Sumatra and 63% of the natural forest have been lost between 1985 and 2008^51^. Additionally, forest fires linked to the ENSO events, and open burning of forest land for agricultural purposes destroy millions of hectares of land in Indonesia on an annual basis, and Riau is often one of the worst impacted areas, owing in part to its high concentration of peatland^52^. We thus infer that the area of occupancy, extent of occurrence and quality of habitat of *P. percura* have declined to such an extent that the population size has reduced by ≥80% over the last three generations since 1989 (30 years approximately; see Nijman and Manullang^53^ for the closely-related *P. melalophos*), thus fulfilling the IUCN criteria for Critically Endangered (A2cd A3cd A4cd).

*Presbytis robinsoni* ranges from northern Peninsular Malaysia through southern Thailand to southern Myanmar. There are no population estimates (neither recent nor in the past), but some of the species’ habitat continues to be converted for agriculture (primarily oil palm) and it is also targeted by the illegal pet trade. Overall, it cannot be evaluated based on population size and/or the restricted population criterion, but *P. robinsoni* is certainly a taxon of conservation concern and is here considered Near Threatened.

### An urgent need for molecular data for additional *Presbytis* populations

**Figure 5:**
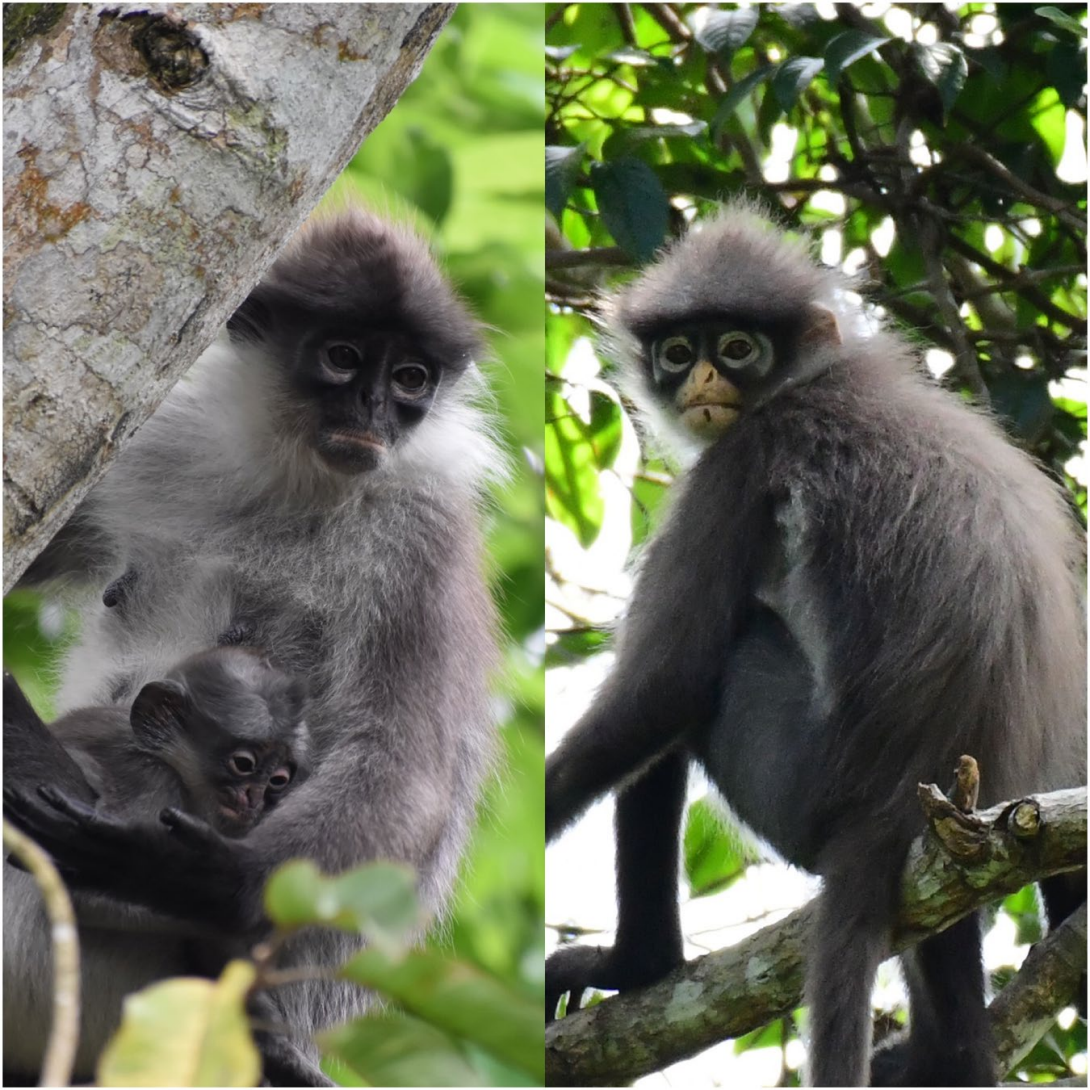
*Presbytis siamensis siamensis* (left image: Lee Zan Hui) and *P. s. cana* (right image: Andie Ang).

**Figure 6:**
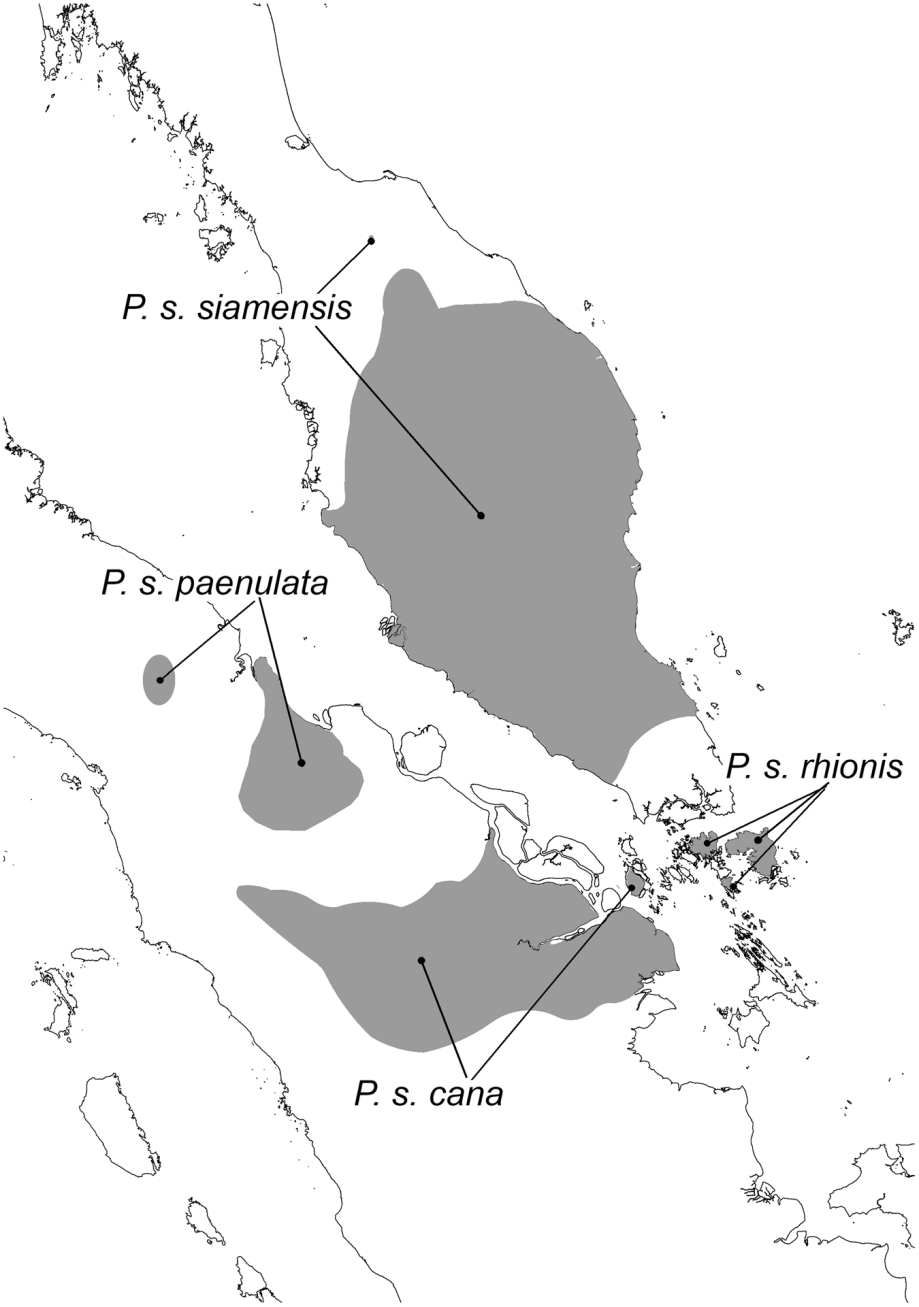
Distribution of four subspecies of *Presbytis siamensis*. Image: Lee Zan Hui.

Our results highlight the need for sampling multiple populations of *Presbytis* species and subspecies because even our limited fieldwork already provided strong evidence for the widespread presence of cryptic diversity or inappropriate synonymization within the genus. Additional data are also needed in order to be able to precisely assign samples and understand the distribution of *Presbytis* species. We collected one faecal sample that was suspected to come from an individual of *P. siamensis* cf. *cana*. However, its placement in the phylogeny reveals that it belongs to a genetically distinct lineage that is more closely related to *P. melalophos* + *mitrata* than *P. s. siamensis* (Supplementary Fig. S2). If the sample was indeed from *P. s. cana*, then the taxonomy of the pale-thighed langur *P. siamensis*, which currently comprises four subspecies, needs to be revisited unless the unexpected signal is due to introgression via the hybridization of two species. Regardless of the explanation, the taxon represented by the sample deserves species status, but the correct scientific name and range remain unclear because *P. s. paenulata* and *P. s. rhionis* lack molecular data. Even if the faecal sample originated from individual of *P. melalophos/mitrata*, its genetic distinctness suggests that these species require more attention. In addition, it would mean that the geographic ranges of these species need revision because the species are unknown from the place of sample collection. Overall, either explanation is reason for concern. Geographically, only *P. s. siamensis* (Fig. 5; left photo) has a wide distribution on the Malay Peninsula while the remaining three subspecies have narrow distributions (Fig. 6). *Presbytis s. cana* (Fig. 5; right photo) occurs in eastern Sumatra and on Kundur Island, *P. s. paenulata* is found mainly in a small-wedge of coastal forest in east-central Sumatra, and *P. s. rhionis* has only been found on the islands of Bintan and Batam (but may also be found on Galang Island) in the Riau Archipelago^11^. Given the extensive habitat loss to oil palm plantations in Sumatra and large-scale economic development in Bintan and Batam, these taxa are likely highly threatened. Study is urgently needed and we submit that faecal samples would be the best way to rapidly address the species limits and distributions. Particular attention should be given to sampling additional *P. s. cana* given the results of this study. Currently, we lack genetic data, even COI barcodes, for many subspecies of *Presbytis* and their distributions are poorly understood. Clearly, Southeast Asian langurs have received insufficient attention and broad surveys are needed that estimate population sizes while collecting faecal samples for a re-assessment of species boundaries^54^.

### Mitochondrial phylogeny of *Presbytis*

In the process of delimiting species, we re-examined the phylogenetic relationships within *Presbytis* by combining the data for mitochondrial genomes with the data generated by Meyer *et al*.^12^ for cyt-b and d-*loop*-HV1. This led to the resurrection of *P. femoralis* and *P. percura* which are here revealed to be sister species and more closely related to several other *Presbytis* species from Sumatra (and *P. rubicunda* in Borneo) than *P. robinsoni* from the Malay Peninsula. Note, that this placement of *P. femoralis* is in conflict with relationships proposed by Abdul-Latiff *et al*.^28^, who obtained a clade comprising of *P. femoralis* + *P. robinsoni* + *P. siamensis* which was sister species to the *Presbytis* species from Sumatra + *P. rubicunda*.

One limitation of our study is the lack of nuclear data for species delimitation and reconstructing relationships (e.g. Wang *et al*.^39^). Unfortunately, obtaining nuclear data from faecal samples remains challenging partially due to the low concentration of primate DNA in faecal samples. We assessed the primate nuclear DNA content in these metagenomes and found that it was only 0.09–3.13% of total DNA (Table 5). However, we would argue that the lack of nuclear data does not seriously challenge our conclusions. Firstly, we reveal deep mitochondrial splits of >2 million years between *P. femoralis* and *P. percura*. Secondly, our proposed species limits are consistent with morphological data that allow for assigning specimens unambiguously to one of the lineages that are here resurrected as species. Lastly, whatever limited information is available for Asian colobines does not point to widespread conflict between nuclear and mitochondrial signals. Wang *et al*.^39^ presents phylogenetic reconstructions based on mitochondrial and nuclear markers for Asian colobines. Four of the six congeneric nodes are congruent between nuclear and mitochondrial data although they came from different individuals (i.e., study used data from multiple sources). Nonetheless, it will be important to develop new approaches for obtaining nuclear data from faecal samples (see Chiou and Bergey^55^).

**Table 5:** Estimation of host DNA content in faecal metagenomes.

Sample ID	% host	% host after removal of human reads
ESBL_1b	0.51	0.49
ESBL_5	0.62	0.59
ESBL_6a	0.23	0.21
ESBL_7	2.00	1.95
ESBL_8b	3.15	3.13
Pres_2	0.11	0.09

### Non-invasive samples for species discovery

Seventeen (61%) of the 28 taxa of *Presbytis* are threatened (Vulnerable, Endangered, or Critically Endangered) while five (18%) taxa are Data Deficient^5,11^. Many taxa continue to be affected by habitat loss. This means that there is an urgent need to resolve species limits in order to better assess their conservation status and needs. Molecular data play a critical role in resolving species limits, and here faecal samples are particularly valuable because they allow for accelerating data collection. Yet, our literature survey suggests that faecal DNA remains underutilized for taxonomic research with only two other published studies explicitly using faecal DNA as evidence for justifying decision on species status: nine members from a brown lemur complex were given species rank^34^ and two subspecies of otters were elevated to species^33^. Faecal samples yield DNA that are valuable not only for taxonomic purposes but also population genetics, diet analyses, microbiome and parasite research, and should be routinely collected during field surveys. In groups such as *Presbytis*, faecal samples would be particularly useful as these animals are shy and many populations are highly threatened.

## Conclusions

We here demonstrate the value of non-invasive faecal samples for addressing taxonomic questions that are of significant conservation importance. Based on mitochondrial DNA (mitogenomes, cyt-b and d-*loop*), we resurrect three species within the *Presbytis femoralis* group. The new species limits also led to a change in the conservation status of *P. femoralis* and *P. percura* which now have to be considered Critically Endangered. We further urge researchers to include the collection of non-invasive faecal samples into their field protocols^8^.

## Materials and Methods

### Literature survey

In order to assess how frequently faecal samples have been used for addressing taxonomic questions, we conducted a survey of mammalian literature for the last 41 years (between 1978–2018). We downloaded the records pertaining to Supertaxon Mammalia (ST = Mammalia) from *Zoological Record* (articles only). We then identified a subset containing valid mammalian species names by using the checklist by Burgin *et al*.^56^. This was done by searching the binomial name of 6,399 species in the list. We also searched for the genus and species names separately to include records that utilize genus abbreviations. We then retrieved the studies that involved DNA/molecular work on faecal samples by searching for the terms (feces/faeces/fecal/faecal/scat/scats) and (DNA/barcod/sequenc/molecul/genom/genetic/microsatellite). We lastly retrieved the records that were classified under Systematics/Taxonomy in the “Broad terms” field and examined the records manually.

### Sample collection, DNA extraction, and sequencing

This study involved two types of datasets: (1) metagenomic data newly generated for faecal samples collected in Sumatra for *Presbytis femoralis percura* and one sample likely to belong to *P. siamensis cana* (herein referred as *P. s*. cf. *cana*) and (2) metagenomic data previously sequenced as part of Srivathsan *et al*.^10^ and reanalysed here because this study did not reconstruct multiple mitochondrial genomes.

Five faecal samples were collected for *P. femoralis percura* during an eight-day (25 April - 2 May 2018) survey in Riau Province^50^. We also collected one faecal sample believed to come from *P. s. cana* as the monkeys were seen on the same tree below which the fresh faecal sample was found. The samples were preserved following a two-step ethanol-silica method^57^ and subsequently stored at a −20 °C freezer at the Andalas University in Sumatra. Genomic DNA was extracted at Andalas University from 50 mg of faeces using QIAamp Fast DNA Stool Mini Kit (QIAGEN, Singapore). DNA was recovered in 30 μl of elution buffer (instead of 200 μl) in order to obtain a higher concentration of DNA. Each sample was also extracted 2–3 times and later pooled to recover more genomic DNA. Genomic DNA of these six samples were sent from Andalas University for Illumina HiSeq X (Illumina Inc., San Diego, CA) sequencing (150 PE) by a commercial provider (NovogeneAIT). A library was constructed for each faecal sample (fragment size 350 bp) using NEBNext Ultra II DNA Library Prep Kit. The five faecal samples for *P. f. percura* and one faecal sample of *P. s*. cf. *cana* were sequenced using HiSeq.

### Bioinformatics for obtaining mitochondrial genomes

Raw reads generated for *P. f. percura* and *P. s. cana* in this study as well as those for six samples of *P. f. femoralis* from Srivathsan *et al*.^10^ were trimmed using Trimmomatic v.0.33^35^ under the following parameters: LEADING:3 TRAILING:3 SLIDINGWINDOW:4:15 MINLEN:50, and the ILLUMINACLIP parameter was set at 2:30:10^35^. New mitochondrial reference genomes were assembled for *P. f. percura and P. s. cana* using MITObim v.1.9^58^ using the available mitogenome of *P. f. femoralis* (KU899140: 16548 bp) from one sample each (ESBL1b and Pres2). Any redundancy due to the circular nature of mitochondrial genome was removed in the resulting assembly (details in readme file in Supplementary Material 3). We then obtained one mitochondrial genomes per faecal sample by mapping the quality-trimmed reads for the sample to the reference genome (ESBL samples to mitochondrial genome from ESBL1b, Pres2 to Pres2 and BLM1–6 to KU899140 or BLM5). Reads were mapped using Bowtie2^59^ under paired end and --end-to-end mode. The resulting SAM files were converted to BAM files using SAMtools^60^. Variants were detected using LoFreq^61^ using parameters similar to Isokallio and Stewart^62^ with a few more stringent criteria: we included mapping quality in LoFreq’s model and also retained base-alignment quality. We furthermore applied a minimum allele frequency of 0.2 while filtering the vcf to accept heteroplasmy only if it was at a frequency of >0.2. Alternate mitochondrial genomes were reconstructed from the resulting vcf file using gatk^63^ FastaAlternateReferenceMaker and heteroplasmic sites were modified as ambiguous nucleotides using custom script. All mitochondrial genomes were annotated using MITOS^64^.

Lastly, we checked the BAM files for each mitochondrial genome for contamination with human reads. The reads were retrieved from BAM file corresponding to each mitochondrial genome and mapped back to reference human mitochondrial genome (NC_012920.1) using Bowtie2 under (--end-to-end, single end). No reads matched with 0 mismatches, a maximum of one read mapped with 1 bp mismatch across the datasets, while the remaining few reads (~3%) mapped at > = 2 bp. In order to assess if these reads were more similar to a *Presbytis* mitogenome than to a human mitogenome, we matched the sequences mapping to the human mitogenome against both human and *P. f. robinsoni* (DQ355299 or NC_008217.1) genomes using BLASTN and retained the best matching sequence. The *P. f. robinsoni* genome selected here is a curated RefSeq genome. Overall 0.06% of the mitochondrial reads preferentially matched to the human genome. The reference genomes for the three subspecies ESBL1b, Pres2 and BLM5 had 1/2874, 0/1054 and 13/22796 reads that were more likely human than Presbytis. In order to ensure that these reads did not affect the reconstruction of the reference mitochondrial genomes, we eliminated the reads from the BAM files using PICARD tools and recalled the consensus sequence as described before. The consensus sequence remained unchanged. The remaining samples also revealed negligible human contamination. ESBL 5, 6a, 8b (*P. f. percura)* and Pres2 (*P. siamensis cf. cana*) had no contaminating signals, while the number of reads for the shorter 76 bp datasets of *P. f. femoralis* revealed the following number of possible contaminating reads: BLM1: 2/4764, BLM2: 4/4307, BLM3: 1/3282, BLM4: 8/8220, BLM6: 3/2781. Overall, the mitochondrial genome reconstructions were not affected.

### Phylogenetic reconstructions and species delimitations

The newly reconstructed mitochondrial genomes were combined with publicly available data from GenBank. For the latter, we downloaded all the mitochondrial sequences for colobine primates and then retained only the data for Asian colobines. We then curated the GenBank records by consulting the source publication and assessing the locality information in order to update the taxonomic names given that many subspecies are now considered species. We excluded those sequences for which the source information was incomplete. This curated set of sequences was used for downstream distance based (Automated Barcode Gap Discovery, ABGD^37^ and Objective Clustering^38^ and tree-based species delimitation analyses (Poisson Tree Processes or PTP)^36^. We also included data for d-*loop* HV1 obtained by Abdul-Latiff *et al*.^28^ for *P. f. femoralis, P. f. robinsoni* and *P. s. siamensis* from Malaysia. Given that these sequences were not submitted to GenBank, we used Nijman’s^26^ reconstruction of the sequences based on a table that lists all variable sites relative to a reference sequence.

For analyses with PTP, we used three datasets: (1) The Asian colobine mitogenome dataset based on genomes for Asian colobines (minimum length > 10,000 bp). The sequences for the 13 mitochondrial CDS, two ribosomal genes, and complete d-*loop* sequences were extracted, aligned, and concatenated. Here, *Colobus guereza* and *Macaca sylvanus* were selected as the outgroups. (2) A second dataset included the *Presbytis* mitogenome+cyt-b + HV1 dataset. This dataset covers more samples because *Presbytis* has been well sampled for cyt-b and the hypervariable region I (HV1) of d-*loop* (see Meyer *et al*.^12^). For the analyses of this dataset, we used *Trachypithecus obscurus* and *T. barbei* as outgroups. (3) The last dataset comprised HV1 sequences only (*Presbytis* HV1-only dataset). This included HV1 sequences obtained by Abdul-Latiff *et al*.^28^ for *P. f. femoralis, P. f. robinsoni* and *P. s. siamensis*.

All coding sequences were aligned in MEGA X^65^ based on amino acid translations (using Clustal). The ribosomal genes and d-*loop* sequences were aligned using MAFFT LINSI^66^. We ensured that only distinct haplotypes were retained and only used the longest sequence if identical sequences were found. The alignments were concatenated in SequenceMatrix 1.7.8^67^. Maximum Likelihood reconstructions were carried out using RAxML v8^68^. For the mitogenome and the *Presbytis* mitogenome+cyt-b + HV1 datasets, we determined best partitioning scheme by providing 42 different partitions to PartitionFinder^69^ corresponding to codon position for the 13 coding regions and 3 separate partitions for 12 S, 16 S and d-*loop*. RAxML was run using GTRGAMMA with the resulting partitioning scheme (no partitioning was done for the HV1 dataset) with 20 independent searches for the best tree. Multiparametric bootstrapping was conducted applying the automatic bootstopping criterion (autoMRE). The resulting trees were subjected to PTP-based species delimitation after excluding outgroups.

Distance-based species delimitation utilized ABGD based on uncorrected distances^70^. We assessed species delimitations under different parameters by varying the slope X = 0.1, X = 0.5 and X = 1 (X = 1.5 was not applicable to the dataset) and prior intraspecific divergences. Species delimitation was carried out based on the Asian colobine mitogenome dataset described above and the *Presbytis* cyt-b and HV1 alignments. The same datasets were also clustered using Objective Clustering as implemented in Species Identifier (Taxon DNA 1.6.2)^38^ at genetic distances of 2.0, 3.0 and 4.0%.

### Divergence dating

Divergence dates between Asian colobine lineages were determined using BEAST v 2.6.0^71^. We used Asian colobine mitogenome dataset but excluded d-*loop* for this analysis due its differing mutational patterns as done for previous studies^12,72^. For divergence estimates, we included the following genomes to the Asian colobine mitogenome dataset: *Pongo pygmaeus* (NC_001646), *Pan troglodytes* (NC_001643), *Homo sapiens* (NC_012920), *Chlorocebus aethiops* (NC_007009), *Macaca sylvanus* (NC_002764), *Papio hamadryas* (NC_001992), *Theropithecus gelada* (NC_019802). We also did a second analysis for *Presbytis* cyt-b as done by Meyer *et al*.^12^ and used a similar strategy for the various steps. Here, representative sequences from different Asian colobine genera were included: *Trachypithecus obscurus* (NC_006900), *Nasalis larvatus* (NC_008216), *Rhinopithecus avunculus* (NC_015485), *Semnopithecus vetulus* (NC_019582) in addition to the above-mentioned sequences for fossil-based calibration. The fossil calibration dates used in this study also followed the dates used by Meyer *et al*.^12^. For mitochondrial genomes, we analysed the data using the following partitioning schemes: by codon (5 partitions: 1,2,3 codon for coding genes, 12 S, 16 S) and by gene. We again tested partitioning by both gene and codon, but found the Effective Sample Size (ESS) to be low for multiple parameters. For cyt-b dataset, we used the 1 + 2 and 3 codon partitioning scheme^12^.

Divergence estimates were based on a relaxed log normal clock and a Yule prior. Site models were unlinked across partitions, and a model-averaging approach was used as implemented in bModelTest^73^. Two independent runs were conducted with 25 million generations with sampling at every 1000 generations. Tracer v 1.7.1 was used to assess convergence, LogCombiner 2.6.1 was used to combine the results and 10% burn-in removal was applied. TreeAnnotator v 2.6.0 was used to summarize the trees.

### Estimation of host DNA content in faecal metagenomes

In order to estimate the amount of host nuclear DNA in the faecal metagenomes, we mapped the metagenomic reads to a colobine reference genome (*Rhinopithecus roxellana*: GCF_000769185.1) using bowtie-2 (--end-to-end and--very-sensitive mode). We next excluded reads that could represent contamination with human DNA. For this, the mapped reads were retrieved from the resulting bam files using samtools. These were mapped back to a combined reference dataset of *R. roxellana* and human genome (GCF_000001405.39, GRCh38) using Bowtie2. Resulting BAM file was filtered to exclude reads with any mismatches, and we then excluded all sequences that matched to human genome only.

### Ethics statement

We followed the Code of Best Practices for Field Primatology (2014). All genetic material were obtained non-invasively through faecal samples; no animals were harmed in the process. We followed the rules and regulations of the Government of Indonesia and RISTEKDIKTI (research permit no. 3051/FRP/E5/Dit.KI/IX/2018).

## Supplementary information

Supplementary Material.

Supplementary Material 2.

Supplementary Material 3.

## Data Availability

The mitochondrial genomes generated during the current study are available in GenBank with accession numbers MN496088-MN496097, and raw data has been submitted to NCBI SRA (PRJNA574841). The Supplementary Materials 2 and 3 contain the BAM files and the GenBank submission.
