# Supplementary material for: Faecal DNA to the rescue: Shotgun sequencing of non-invasive samples reveals two subspecies of Southeast Asian primates to be Critically Endangered species
Source: Sci Rep. 2020 Jun 10;10:9396. doi: 10.1038/s41598-020-66007-8 (PMC7287133; doi:10.1038/s41598-020-66007-8)
Supplement: Supplementary file 1 — Supplementary Material. [file 41598_2020_66007_MOESM1_ESM.pdf]

Faecal DNA to the rescue: Shotgun sequencing of non-invasive samples reveals two subspecies of Southeast Asian primates to be Critically Endangered species

Andie Ang[#], Dewi Imelda Roesma[#], Vincent Nijman[3], Rudolf Meier[4], Amrita Srivathsan[*], Rizaldi[*]

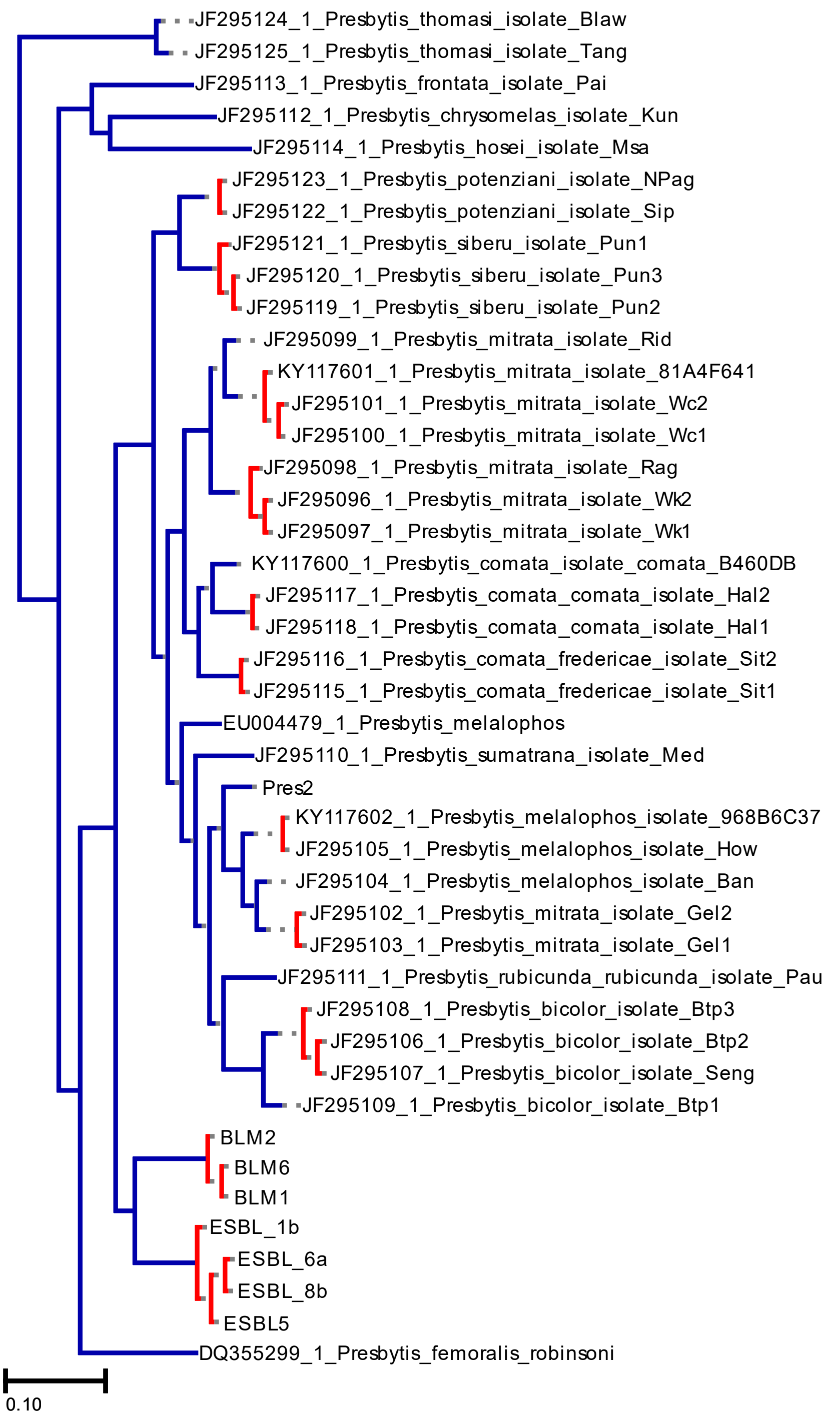

Figure S1: Species delimitation using PTP based on *Presbytis* mitogenome+Cytb+ HV1 dataset

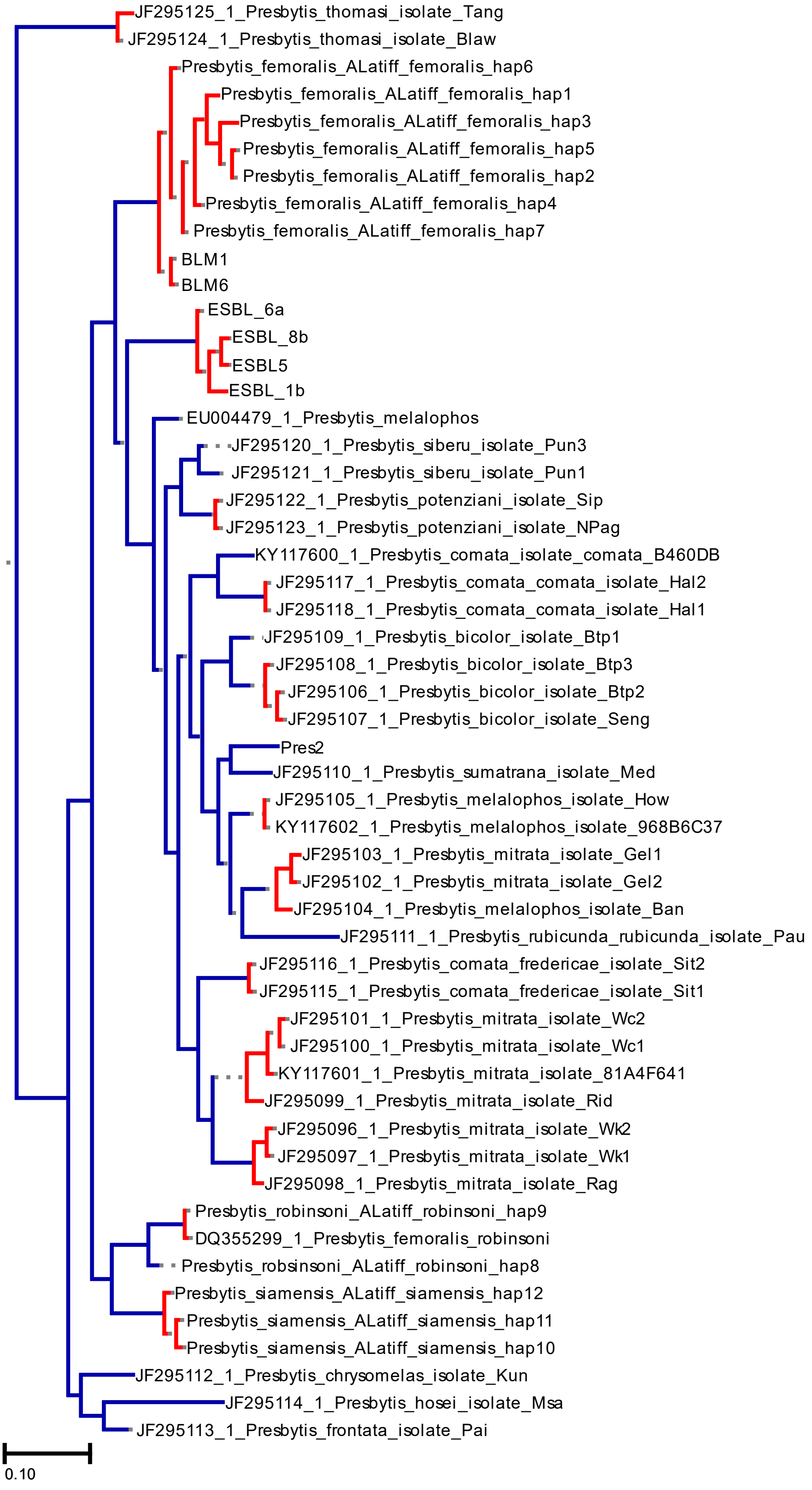

Figure S2: Species delimitation using PTP based on *Presbytis* HV1 only dataset

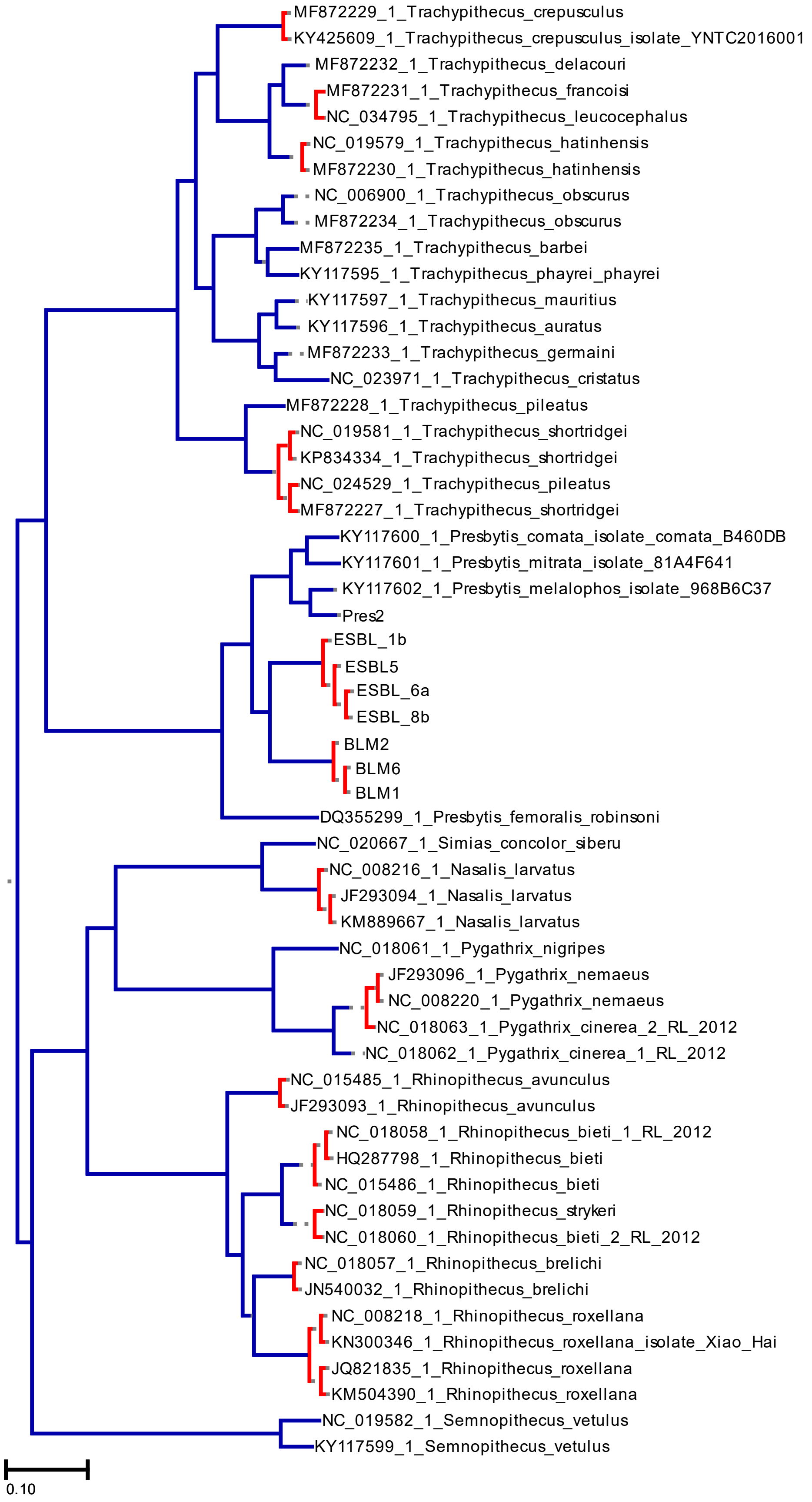

Figure S3: Species delimitation using PTP based on Asian colobine mitogenome dataset

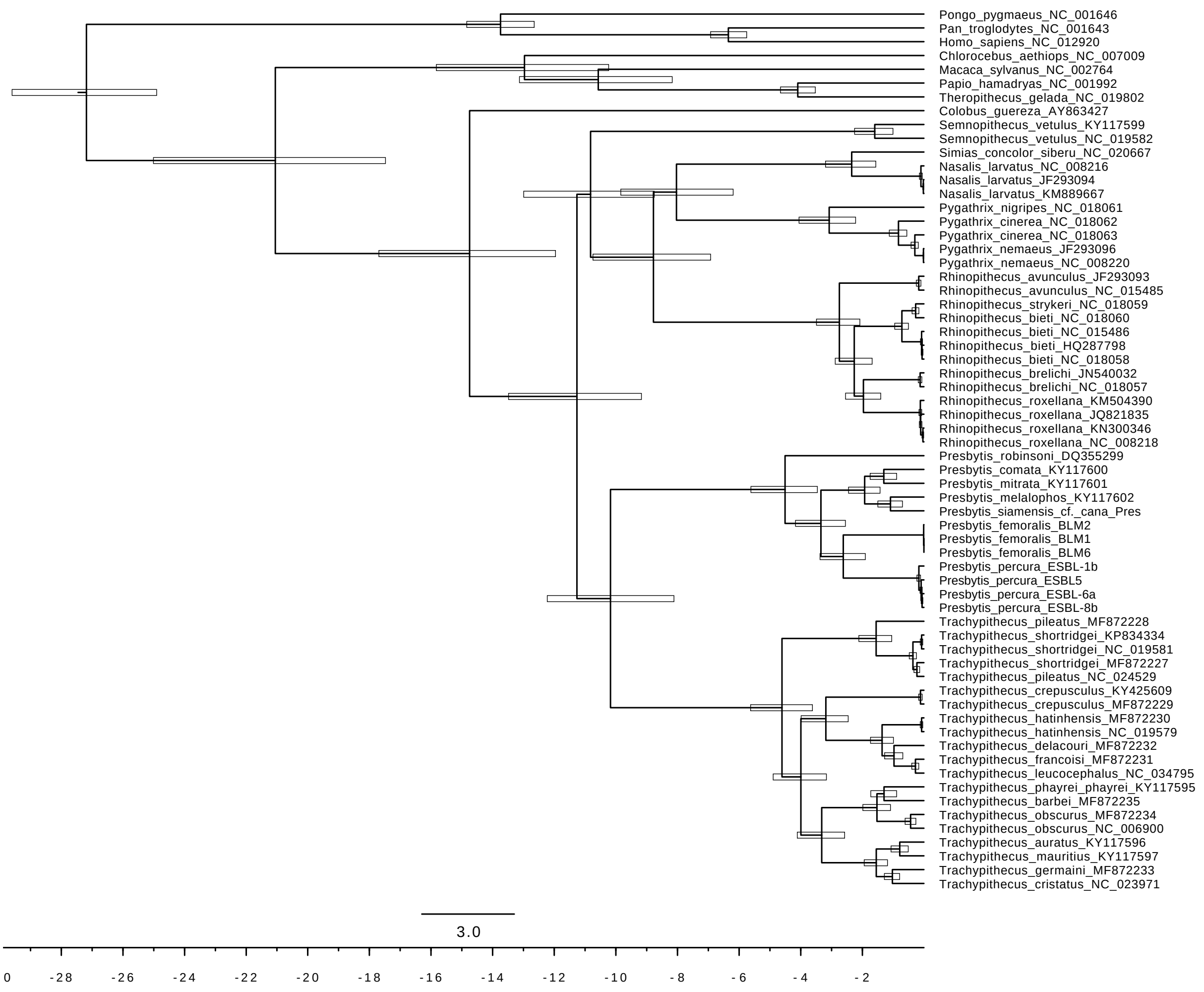

Figure S4. Divergence times estimates based on mitochondrial genomes for Asian colobine primates (partitioned by gene)

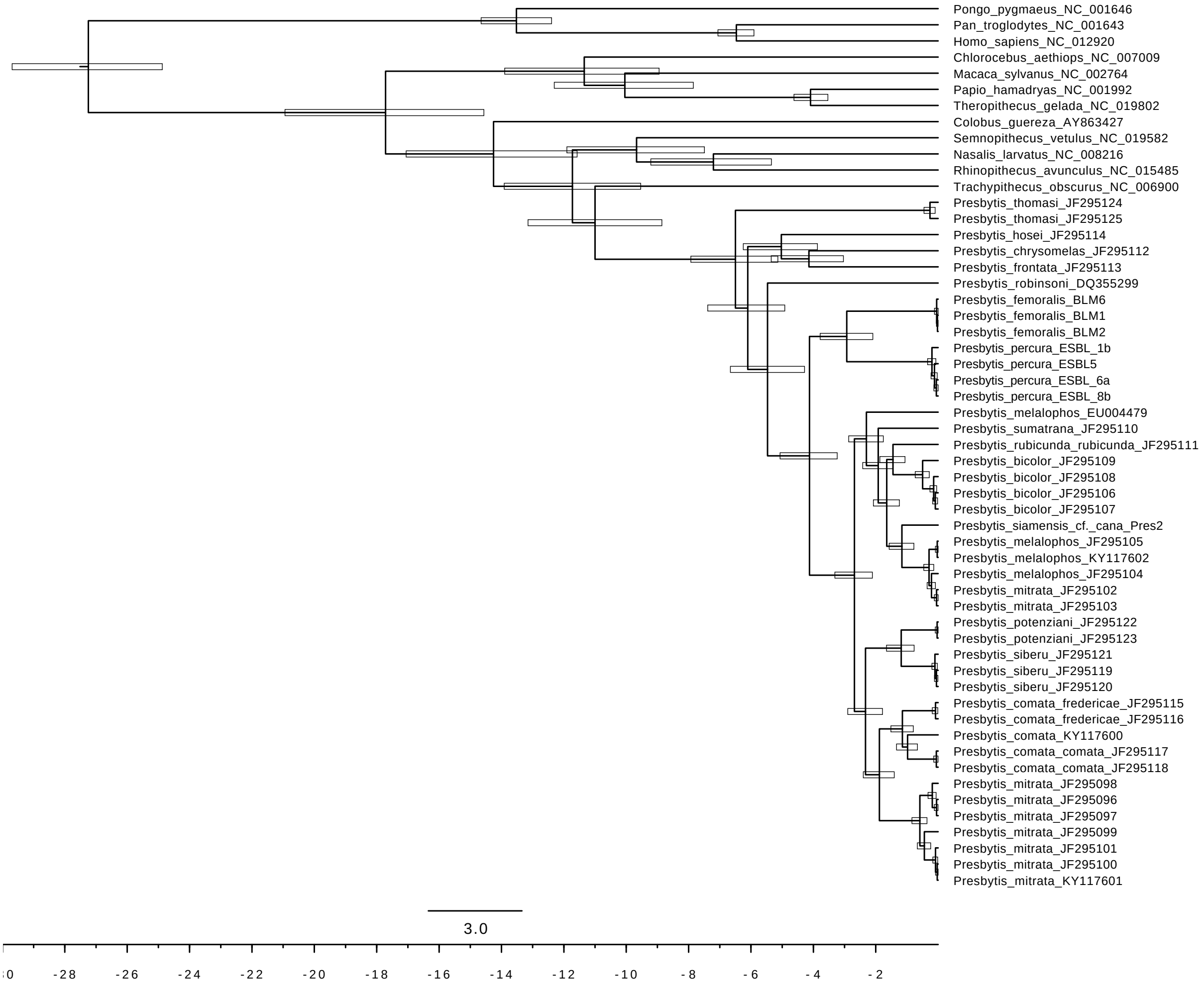

Figure S5 Divergence times estimates based on cyt-b for *Presbytis*.
